# Associations of genetic liability for Alzheimer’s disease with cognition and eye movements in a large, population-based cohort study

**DOI:** 10.1038/s41398-022-02093-8

**Published:** 2022-08-19

**Authors:** Annabell Coors, Mohammed-Aslam Imtiaz, Meta M. Boenniger, N. Ahmad Aziz, Ulrich Ettinger, Monique M. B. Breteler

**Affiliations:** 1 Population Health Sciences, German Center for Neurodegenerative Diseases (DZNE), Bonn, Germany; 2 Department of Neurology, Faculty of Medicine, University of Bonn, Bonn, Germany; 3 Department of Psychology, University of Bonn, Bonn, Germany; 4 Institute for Medical Biometry, Informatics and Epidemiology (IMBIE), Faculty of Medicine, University of Bonn, Bonn, Germany

**Keywords:** Predictive markers, Diseases, Clinical genetics

## Abstract

To identify cognitive measures that may be particularly sensitive to early cognitive decline in preclinical Alzheimer’s disease (AD), we investigated the relation between genetic risk for AD and cognitive task performance in a large population-based cohort study. We measured performance on memory, processing speed, executive function, crystallized intelligence and eye movement tasks in 5182 participants of the Rhineland Study, aged 30 to 95 years. We quantified genetic risk for AD by creating three weighted polygenic risk scores (PRS) based on the genome-wide significant single-nucleotide polymorphisms coming from three different genetic association studies. We assessed the relation of AD PRS with cognitive performance using generalized linear models. Three PRS were associated with lower performance on the Corsi forward task, and two PRS were associated with a lower probability of correcting antisaccade errors, but none of these associations remained significant after correction for multiple testing. Associations between age and trail-making test A (TMT-A) performance were modified by AD genetic risk, with individuals at high genetic risk showing the strongest association. We conclude that no single measure of our cognitive test battery robustly captures genetic liability for AD as quantified by current PRS. However, Corsi forward performance and the probability of correcting antisaccade errors may represent promising candidates whose ability to capture genetic liability for AD should be investigated further. Additionally, our finding on TMT-A performance suggests that processing speed represents a sensitive marker of AD genetic risk in old age and supports the processing speed theory of age-related cognitive decline.

## Background

Alzheimer’s disease (AD) can roughly be divided into three clinical disease stages: a pre-symptomatic phase characterised by pathological brain changes, a prodromal phase characterised by subtle cognitive impairment and then lastly the dementia stage in which impairments occur in multiple domains and lead to loss of function [1]. As only 10–15% of individuals with amnestic mild cognitive impairment (MCI) develop AD each year [2], prediction of disease progression is of great interest to identify those individuals best suited for disease-delaying interventions, such as drug trials [3]. A meta-analysis found that particularly episodic verbal memory performance (e.g., delayed recall of a word list) and performance in language tasks that implicate semantic memory and executive function (e.g., the word fluency task) have high predictive accuracy for disease progression [4].

Eye movement assessment may be an alternative promising method to identify individuals at high risk for AD as it provides language-independent and culture-fair measures [5, 6] of multiple cognitive, perceptual and motor processes, including attention, processing speed, motion processing, working memory, learning and inhibition [7, 8]. In people with AD, instability of fixation [9–11] and deficits in the prosaccade [9, 12–14], antisaccade [12–16] and smooth pursuit tasks [16, 17] have been reported.

In the prosaccade task, participants are asked to perform a saccade, i.e., a rapid eye movement executed to bring an object of interest onto the fovea, towards a sudden-onset peripheral target. Prosaccade tasks measure overt attention and response speed [18], and individuals with AD were found to have longer latencies, i.e., longer reaction times for the initiation of a saccade towards the peripheral target, compared to healthy controls [12–14]. The antisaccade task has the same task design as the prosaccade task but participants are asked to execute their first saccade in each trial in the opposite direction of the peripheral target [19]. In this task, which is a good measure of inhibitory control [20], individuals with AD and MCI have consistently been found to make more direction errors compared to controls, i.e., a first saccade within a trial towards the target instead of towards its mirror position [12, 14]. Additionally, they are also less likely to correct their direction errors than controls [13, 15, 16]. For antisaccade latencies, the majority of studies reported higher latencies in AD [13, 14]. Moreover, in both saccade tasks, individuals with AD and amnestic MCI were found to perform hypometric saccades, which is reflected by a low value in the spatial accuracy measure called amplitude gain (amplitude of the eye movement divided by the amplitude of the target movement) and is accompanied by a high spatial error [9, 14, 21]. Research on whether saccade velocity and antisaccade costs (antisaccade latency minus prosaccade latency) differ between individuals with AD and healthy controls is still largely lacking [14], but some evidence suggests that performance in these measures may also be impaired in AD [13, 21].

Smooth pursuit eye movements (SPEMs) are performed to keep a slowly moving object on the fovea [22]. SPEMs have been found to have a lower velocity gain (ratio of eye velocity to target velocity) in AD [16, 17].

Importantly, performance in many oculomotor measures has been found to correlate with dementia severity, for example, instability of fixation [11, 23], prosaccade and antisaccade latency and amplitude gain [11, 21, 24], antisaccade direction error rate [25, 26] and correction rate [24]. Additionally, some studies have found that oculomotor performance may also help to differentiate between individuals with amnestic MCI and non-amnestic MCI [24, 27]. This may be relevant for predicting disease progression to AD, as individuals with amnestic MCI seem to be more likely to develop AD than individuals with non-amnestic MCI [28]. However, the usefulness of eye movements in identifying individuals at high risk for AD remains largely unexplored.

Genetic factors play a substantial role in the development of AD [29]. Polygenic risk scores (PRS) for AD, which represent the weighted sum of AD risk alleles that an individual carries, are well-suited to quantify genetic risk for AD as they account for the complex polygenic nature of AD [30].

Studies on the association between AD PRS and performance in classical cognitive tests have been conducted in samples including mainly older individuals without dementia or a mixture of individuals with MCI and individuals without dementia. AD PRS have been found to be significantly associated with both baseline episodic verbal memory performance [31–33] and longitudinal decline in episodic verbal memory [32–35], yet other studies could not confirm these findings in individuals with MCI or healthy participants [36, 37]. AD PRS were not associated with baseline working memory in most studies [33, 37], although working memory was found to deteriorate faster with higher PRS [34, 35]. Similarly, AD PRS were not associated with baseline performance in processing speed in several population-based studies [31, 33], except for a subgroup of 70 to 99-year-olds [31]. However, AD PRS were related to decline in processing speed [34, 35]. Studies examining the relation between AD PRS and baseline executive function either reported negative [31] or no associations[32, 37], whereas studies exploring the relation between AD PRS and longitudinal change in executive function were inconclusive [38, 39]. We are only aware of one study that investigated the relation between genetic risk for AD and eye movement performance [18]. That study found that antisaccade performance was similar between apolipoprotein E (*APOE*) ε4 carriers and non-carriers, yet APOE ε4 carriers performed worse on the prosaccade task. However, the sample size was small (*N* = 97), the participants were relatively young (17–35 years) and AD genetic risk was only based on *APOE* ε4 carrier status [18].

Here, we aimed to assess which, if any, cognitive measures are sensitive to genetic susceptibility for AD in a large, population-based sample including a wide age range. We investigated the relation with both classical tests of cognitive function and eye movement performance. Additionally, we investigated whether genetic liability for AD modifies the association between age and cognitive performance.

## Materials and methods

### Participants

We used baseline data from the Rhineland Study, a community-based cohort study that includes inhabitants aged ≥30 years (current age range: 30 to 95 years) from two geographically defined areas in Bonn, Germany. The only exclusion criterion is not having sufficient command of the German language to provide written informed consent. The ethics committee of the Medical Faculty of the University of Bonn approved the study that was carried out in accordance with the recommendations of the International Council for Harmonisation Good Clinical Practice standards (ICH-GCP). Of originally 5801 participants who provided blood samples between March 2016 and October 2021, 5189 remained after quality control of genetic data (see Section 2.2). Of those, 5182 had data in at least one cognitive task and were therefore included in the analyses.

### Genetic data and polygenic risk scores

Blood samples were genotyped using Illumina Omni-2.5 exome arrays containing 2,612,357 single-nucleotide polymorphisms (SNPs). Genotype data were processed using GenomeStudio (version 2.0.5) and quality controlled using PLINK software (version 1.9). SNP exclusion criteria were Hardy-Weinberg disequilibrium (*p* < 1*10^−6^), minor allele frequency, (<0.01) and poor genotyping rate (<99%). Participants with poor DNA samples were excluded, which comprised 41 cases with poor call rate (<95%), 86 cases with abnormal heterozygosity, 290 cases with cryptic relatedness and 30 cases with gender mismatch. Because variation in population structure can cause systematic differences in allele frequencies [40], we used EIGENSTRAT (version 16000), which uses principal components to detect and correct for variation in population structure [40] (exclusion of *N* = 165 participants). Finally, we imputed missing SNPs based on the 1000 Genomes reference panel [41] using IMPUTE (version 2) [42]. To include only SNPs with high imputation quality, we checked for an info score metric greater than 0.3 as this value is considered to indicate reliable imputation quality [43].

Using PLINK (version 1.9), we created three different weighted AD PRS scores based on the genome-wide significant SNPs (i.e., those SNPs that had a *p*-value below 5*10^−8^ in the respective genome-wide association study (GWAS)). One PRS (PRS_Jansen_) was created based on 29 genome-wide significant SNPs that were found in the meta-analysis by Jansen et al. in 2019 [44] (https://ctg.cncr.nl/software/summary_statistics; retrieved on January 15, 2021). The two additional PRS scores were created based on the genome-wide significant SNPs identified in two more recent meta-analyses by Wightman et al. [45] (PRS_Wightman_) and Schwartzentruber et al. [46] (PRS_Schwartzentruber_). The study by Wightman et al. [45] is an extension of the study by Jansen et al. [44]. This study included a larger number of participants in one of the included cohorts as well as data from 12 additional cohorts (in total: *N* = 1,126,563 participants), and identified 38 risk loci. However, the authors could only provide us with the beta estimates from the summary statistics excluding the UK biobank (*N* = 364,859) and the 23andMe data (*N* = 363,646). Thus, we compared the signs of the z-scores they had reported for the original data set with those of the beta estimates they had provided and found that they were consistent. Additionally, one SNP (rs115186657) was missing in the summary statistics that they had provided and one SNP (rs2632516) was not available in our data. Therefore, we were able to include 36 SNPs in PRS_Wightman_. To create PRS_Schwartzentruber_, we used all 37 risk loci for AD that were identified in the meta-analysis by Schwartzentruber et al. [46]. This meta-analysis combined the data of the study by Kunkle et al. from 2019 [47] and the updated results of a GWAS study of UK Biobank participants with a family history of AD. Earlier results from the GWAS analysis of the UK Biobank AD proxy cases were also included in the Jansen et al. publication [44]. PRS_Wightman_ and PRS_Schwartzentruber_ were highly correlated with each other (Pearson’s *r* = 0.95) but their correlations were lower with PRS_Jansen_ (PRS_Wightman_: *r* = 0.60; PRS_Schwartzentruber_: *r* = 0.63). This may be due to the fact that the two more recent GWAS only partially replicated the genome-wide significant loci reported by Jansen et al. [44] (Schwartzentruber: replication of 23 loci out of 29 from Jansen; Wightman: replication of 22 loci out of 29 from Jansen).

### Outcome measures

We measured cognitive performance using classical tests of working memory, episodic verbal memory, processing speed, executive function and crystallized intelligence, along with an eye movement test battery. The examinations were administered following a standardized procedure by certified study technicians. Working memory was assessed with the forward and backward digit span task and the forward and backward Corsi block-tapping test (Corsi), adapted from the PEBL battery [48]. The Auditory Verbal Learning and Memory Test (AVLT) was used to assess episodic verbal memory (immediate recall: sum of correctly recalled nouns in the first five trials, delayed recall: number of correctly recalled words after a time delay of 20 to 30 min) [49]. Processing speed was measured with a numbers-only trail-making test (TMT-A: time to completion). Executive function was assessed with a 60 s categorical word fluency task (number of uniquely named animals) and a number-and-letters trail-making test (TMT-B: time to completion). Crystallized intelligence was measured with the 37-item Mehrfachwahl-Wortschatz-Intelligenztest (MWT-B), a vocabulary test in which participants had to select an existing German word among four non-words in each of 37 trials [50].

The eye movement test battery consisted of fixation, SPEM, prosaccade and antisaccade tasks. For recording of eye movements, we used video-based infrared oculography (EyeLink 1000 and EyeLink 1000 Plus; SR Research Ltd) at 1000 Hz. Fixations were defined as periods of at least 100 ms duration without blinks or saccades directed toward the target (a white circle 0.35° in diameter on black background). The target appeared first in the centre (*x* = 0°, *y* = 0°) for 5 s and then in a random order for 10 s each at the top (*x* = 0°, *y* = 9.63°), bottom (*x* = 0°, *y* = −9.63°), left (*x* = −9.63°, *y* = 0°), or right (*x* = 9.63°, *y* = 0°), always returning to the centre after each of these four eccentric locations. Thus, the central position had to be fixated four times in total. To obtain measures for fixation stability, we calculated the mean spatial error of gaze position (in degree of visual angle), mean saccade rate (saccades/second) and mean blink rate (blinks/second) during fixation. In the 21 s long SPEM task, the target started in the centre and then moved horizontally for ten full cycles in a sinusoidal waveform between ±9.63° at a frequency of 0.5 Hz. All eye movements with velocity <30°/s and duration ≥50 ms were classified as SPEMs. We determined the mean SPEM gain for the middle two quarters of each half-cycle of target motion (left to right or right to left) separately and then took the average of these values to calculate the mean velocity gain (in %). In all tasks, saccades were defined as eye movements with an amplitude >1° and either a velocity ≥60°/s or a velocity ≥22°/s and an acceleration ≥3800°/s^2^. We calculated the mean saccade rate (in saccades/second) during smooth pursuit. Prosaccade and antisaccade tasks consisted of 30 trials each (plus six antisaccade practice trials). In each trial, the target appeared first in the centre for a random duration of 1–2 s (average 1.5 s) and then stepped randomly to the left or right (*x* = ±9.63°, *y* = 0°, 15 times per side), where it remained for 1 s before returning to the centre for the next trial. For both saccade tasks, we calculated mean latencies (in ms), the two spatial accuracy measures amplitude gain and spatial error (both in %), and amplitude-adjusted and unadjusted peak velocities (in degree of visual angle/s) for valid trials with a directionally correct initial saccade. For the antisaccade task, we additionally calculated costs (in ms), direction error rate (in %), and correction rate (the percentage of direction errors corrected by participants who made at least 5 antisaccade direction errors by performing a saccade toward the mirror position that crossed at least the midline) for valid trials. Trials were defined as valid when the fixation on the central fixation point started at least 100 ms before peripheral target onset and was no more than 3° off the fixation point. Further, the initial saccade had to end before the peripheral target timed out and saccades with amplitude <1° or latency <80 ms were excluded. Additionally, no saccade or blink was allowed to occur during this period. To obtain reliable data, there had to be ≥7 valid and correct trials for prosaccade and antisaccade outcomes, except for the direction error and correction rate, for which only the criterion of valid but not correct trials applied. Additionally, for all antisaccade outcomes, at least one corrective saccade had to occur in case of ≥5 direction errors. A more detailed description of the oculomotor data acquisition can be found in a previous publication [51].

### Statistical analyses

Statistical analyses were performed in RStudio (version 1.3.959, R-base version 4.0.3) using a two-sided significance test with an alpha level of 0.05. We assessed the associations between genetic risk for AD and cognitive performance separately for the three different AD PRS scores using multivariable linear or one-inflated beta regression models for each cognitive outcome. Models included z-standardized AD PRS as the predictor variable and were adjusted for age, age^2^ and sex, using mean-centred age to reduce collinearity between the main and quadratic term [52]. In order to correct for population stratification, we additionally adjusted for the first six genetic principal components [40]. We imputed missing covariate data using predictive mean matching (Hmisc package, 10 bootstrap replicates). We report unadjusted and false discovery rate adjusted (FDR-adjusted, *N* = 28 comparisons) *p*-values. We were particularly interested in cognitive outcomes that were consistently associated with all three different PRS to identify the most robust cognitive indicators of genetic risk for AD.

As age is a key risk factor for AD [53], we further examined whether genetic risk for AD modified the associations between age and cognitive outcomes by including PRS*age and PRS*age^2^ in the models and comparing the model fit with a likelihood ratio test. In case of significant interactions, we plotted the association between age and the respective cognitive outcome for three different PRS groups (low: z-standardised PRS score below −1; medium: z-standardised PRS score between −1 and 1; high: z-standardized PRS score above 1) separately to visualize how age interacts with genetic susceptibility to influence cognitive decline. Additionally, we tested differences in the slopes between the three PRS groups using Tukey post-hoc tests (pairs-function of the emmeans package [54] in R).

All models were checked for multicollinearity (variance inflation factor, R package car, vif-function), homoscedasticity (scale-location plot) and normality of residuals (quantile-quantile-plot). Because the normality assumption was violated for performance in TMT-A and TMT-B, prosaccade and antisaccade spatial error, and the three fixation outcomes spatial error, saccade rate and blink rate, we log-transformed those outcome variables. Because severe skewness of performance in antisaccade correction rate could not be reduced by log-transformation, we used a one-inflated beta regression model (gamlss package) instead, which is a mixture model consisting of a logistic regression model and a beta regression model. The logistic regression part of the one-inflated beta regression models whether or not AD PRS is associated with the probability of correcting all versus not correcting all antisaccade direction errors. In a second step, the beta regression model part tests whether AD PRS is associated with the percentage of uncorrected antisaccade direction errors in those individuals who did not correct all of their antisaccade direction errors.

We additionally performed a post-hoc power analysis using G-Power (version 3.1) [55] to evaluate which effect sizes for the associations between AD PRS and cognitive outcomes we would be able to detect with our sample size with a statistical power of between 80% to 90%. For this, we performed an *F*-test with one predictor, setting the sample size to 5182 participants, and the type I error rate to 0.05.

## Results

### Study sample

Sample characteristics are displayed in Table 1. Participants were overall highly educated and only 0.1% reported a diagnosis of AD. The eye movement measure antisaccade correction rate was computed for 3053 participants, representing the number of participants who made at least 5 antisaccade direction errors and corrected at least one of these direction errors. Of these 3053 participants, 677 participants did not correct all of their antisaccade direction errors.

**Table 1:** Sample characteristics.

Number of participants, *N*	5182
Age [years], M (SD)	55.5 (13.8)
30–39 years	800 (15.4)
40–49 years	928 (17.9)
50–59 years	1470 (28.4)
60–69 years	1071 (20.7)
70–79 years	684 (13.2)
80+ years	229 (4.4)
Sex, *N* (%) women	2890 (55.8)
Education level, *N* (%)	5134 (99.1)
High	2789 (54.3)
Middle	2260 (44.0)
Low	85 (1.7)
Diagnosis of Alzheimer’s Disease, *N* (%)	5 (0.1)
*APOE* ε4-carriers (ε4/ε4, ε2/ε4, ε3/ε4), *N* (%)	1326 (25.8)
Working memory	
Digit span forward [number of digits], mean (SD) for *N* = 5109; max = 9	6.4 (1.2)
Digit span backward [number of digits], mean (SD) for *N* = 5102; max = 9	4.8 (1.2)
Corsi forward [number of blocks], mean (SD) for *N* = 4972; max = 9	4.9 (1.1)
Corsi backward [number of blocks], mean (SD) for *N* = 4937; max = 9	4.8 (1.0)
Episodic verbal memory	
AVLT - immediate recall [sum of recalled words over recall 1 to 5], mean (SD) for *N* = 5160; max = 75	51.3 (10.1)
AVLT - delayed recall [number of recalled words], mean (SD) for *N* = 5152 max = 15	10.3 (3.3)
Processing speed	
Trail-making test A [completion time in s], median (IQR) for *N* = 5109	33.2 (15.1)
Executive function	
Trail-making test B [completion time in s], median (IQR) for *N* = 5089	43.9 (26.7)
Word fluency task [number of unique words], mean (SD) for *N* = 5132	26.4 (6.9)
Crystallized intelligence	
MWT-B [sum of correctly recognized words], mean (SD) for *N* = 4886; max = 37	30.6 (3.4)
Fixation performance	
Spatial error (RMSE) [°], median (IQR) for *N* = 4744	0.9 (0.3)
Saccade frequency [N/s], median (IQR) for *N* = 4744	0.2 (0.1)
Blink rate [N/s], median (IQR) for *N* = 4676	0.1 (0.2)
Smooth pursuit performance	
Velocity gain [%], mean (SD) for *N* = 4761	78.1 (16.3)
Saccade rate [N/s], mean (SD) for *N* = 4762	2.2 (0.6)
Prosaccade performance	
Prosaccade latency [ms], mean (SD) for *N* = 4747	190.6 (28.4)
Amplitude gain [%], mean (SD) for *N* = 4747	93.8 (6.7)
Spatial error [%], median (IQR) for *N* = 4747	8.2 (5.3)
Peak velocity [°/s], mean (SD) for *N* = 4747	364.8 (57.6)
Amplitude-adjusted peak velocity, mean (SD) for *N* = 4747	3.9 (0.6)
Antisaccade performance	
Latency [ms], mean (SD) for *N* = 4178	282.0 (50.6)
Amplitude gain [%], mean (SD) for *N* = 4178	112.0 (27.8)
Spatial error [%], median (IQR) for *N* = 4178	26.7 (17.4)
Peak velocity [°/s], mean (SD) for *N* = 4178	346.6 (67.3)
Amplitude-adjusted peak velocity, mean (SD) for *N* = 4178	3.2 (0.8)
Antisaccade costs [ms], mean (SD) for *N* = 4165	91.8 (43.1)
Antisaccade error rate [%], mean (SD) for *N* = 4622	31.6 (23.6)
Antisaccade correction rate [%], median (IQR) for *N* = 3053	100.0 (0)

We indicated the mean and standard deviation for almost normally distributed variables and the median and interquartile range for non-normally distributed variables. Education level was determined using the International Standard Classification of Education 2011 (ISCED) and was coded as low (lower secondary education or below), middle (upper secondary education to undergraduate university level) and high (postgraduate university study).

*N* number of participants, *SD* standard deviation, *IQR* interquartile range, *max* maximum, *AVLT* Auditory Verbal Learning and Memory Test, *MWT-B* Mehrfachwahl-Wortschatz-Intelligenztest.

### Associations between AD PRS and cognitive performance

The associations between AD PRS_Jansen_, PRS_Wightman_, PRS_Schwartzentruber_ and cognitive outcomes are displayed in Table 2.

**Table 2:** Associations between three different Alzheimer’s disease polygenic risk scores (PRS) and cognitive test scores and eye movement outcomes.

Cognitive outcome	b (95%-CI) for AD PRS Jansen	*p*-value	FDR-adjusted p-value	b (95%-CI) for AD PRS Wightman	*p*-value	FDR-adjusted *p*-value	b (95%-CI) for AD PRS Schwartzentruber	*p*-value	FDR-adjusted *p*-value
Outcomes - classical cognitive tasks									
Digit span forward [number of digits]	−0.017 (−0.048, 0.013)	0.273	0.588	0.006 (−0.025, 0.037)	0.693	0.808	0.001 (−0.030, 0.031)	0.968	1.000
Digit span backward [number of digits]	0.016 (−0.016, 0.049)	0.323	0.595	**0.034 (0.001, 0.067)**	**0.041**	**0.383**	0.027 (−0.006, 0.059)	0.105	0.368
Corsi forward [number of blocks]	**−0.031 (−0.058, −0.005)**	**0.022**	**0.252**	**−0.031 (−0.058, −0.004)**	**0.025**	**0.383**	**−0.038 (−0.065, −0.012)**	**0.005**	**0.140**
Corsi backward [number of blocks]	−0.018 (−0.044, 0.008)	0.167	0.585	−0.015 (−0.041, 0.010)	0.242	0.522	−0.014 (−0.040, 0.012)	0.281	0.564
AVLT – immediate recall [sum of recalled words]	−0.147 (−0.371, 0.076)	0.197	0.588	−0.077 (−0.301, 0.147)	0.499	0.665	−0.114 (−0.338, 0.110)	0.318	0.564
AVLT – delayed recall [number of words]	−0.037 (−0.112, 0.039)	0.340	0.595	−0.034 (−0.110, 0.041)	0.375	0.665	−0.038 (−0.114, 0.037)	0.320	0.564
Word fluency task [number of animals]	−0.137 (−0.316, 0.043)	0.137	0.548	−0.104 (−0.283, 0.076)	0.258	0.522	−0.133 (−0.313, 0.047)	0.146	0.409
Trail-making test A [log s]	0.002 (−0.001, 0.006)	0.217	0.588	**0.004 (0.000, 0.007)**	**0.029**	**0.383**	0.003 (−0.001, 0.006)	0.097	0.368
Trail-making test B [log s]	0.003 (−0.001, 0.008)	0.123	0.548	0.004 (−0.001, 0.008)	0.084	0.470	0.004 (−0.001, 0.008)	0.096	0.368
MWT-B [sum of correctly recognised words]	−0.007 (−0.098, 0.085)	0.887	0.920	−0.033 (−0.125, 0.058)	0.474	0.665	−0.037 (−0.129, 0.054)	0.423	0.564
Outcomes - eye movement tasks									
Log of spatial error during fixation [log °]	0.002 (−0.001, 0.006)	0.250	0.588	0.002 (−0.002, 0.006)	0.259	0.522	0.002 (−0.001, 0.006)	0.248	0.564
Log of saccade rate during fixation [log N/s]	−0.001 (−0.006, 0.005)	0.845	0.910	0.001 (−0.004, 0.007)	0.692	0.808	0.002 (−0.003, 0.008)	0.416	0.564
Log of blink rate during fixation [log N/s]	−0.001 (−0.003, 0.000)	0.085	0.548	−0.001 (−0.003, 0.000)	0.143	0.500	−0.001 (−0.002, 0.001)	0.422	0.564
Smooth pursuit velocity gain [%]	−0.145 (−0.553, 0.264)	0.487	0.620	−0.389 (−0.797, 0.019)	0.061	0.427	−0.393 (−0.801, 0.016)	0.059	0.368
Saccade frequency during smooth pursuit [N/s]	**−0.017 (−0.033, −0.002)**	**0.027**	**0.252**	−0.001 (−0.017, 0.014)	0.857	0.908	−0.003 (−0.018, 0.013)	0.715	0.834
Prosaccade latency [ms]	**0.889 (0.177, 1.601)**	**0.014**	**0.252**	0.407 (−0.303, 1.117)	0.261	0.522	0.594 (−0.117, 1.305)	0.102	0.368
Prosaccade amplitude gain [%]	−0.085 (−0.272, 0.101)	0.370	0.599	−0.143 (−0.329, 0.043)	0.132	0.500	−0.178 (−0.364, 0.009)	0.062	0.368
Log of prosaccade spatial error [log %]	0.003 (−0.003, 0.008)	0.300	0.595	0.001 (−0.004, 0.007)	0.669	0.808	0.003 (−0.003, 0.008)	0.335	0.564
Prosaccade peak velocity [°/s]	−0.925 (−2.549, 0.698)	0.264	0.588	0.559 (−1.060, 2.178)	0.499	0.665	0.077 (−1.544, 1.699)	0.926	0.997
Amplitude-adjusted peak prosaccade velocity	−0.006 (−0.022, 0.009)	0.419	0.599	0.011 (−0.005, 0.026)	0.179	0.522	0.007 (−0.009, 0.023)	0.387	0.564
Antisaccade latency [ms]	0.563 (−0.830, 1.956)	0.428	0.599	−0.110 (−1.493, 1.273)	0.876	0.908	0.072 (−1.314, 1.458)	0.919	0.997
Antisaccade amplitude gain [%]	−0.175 (−1.022, 0.672)	0.686	0.768	−0.517 (−1.357, 0.324)	0.228	0.522	−0.654 (−1.496, 0.188)	0.128	0.398
Log of antisaccade spatial error [log %]	0.005 (−0.001, 0.011)	0.133	0.548	−0.002 (−0.008, 0.004)	0.462	0.665	−0.002 (−0.008, 0.004)	0.499	0.635
Antisaccade peak velocity [°/s]	−0.597 (−2.641, 1.447)	0.567	0.690	−0.184 (−2.213, 1.845)	0.859	0.908	−0.506 (−2.539, 1.527)	0.626	0.762
Amplitude-adjusted peak antisaccade velocity	0.010 (−0.014, 0.034)	0.405	0.599	0.018 (−0.006, 0.042)	0.133	0.500	0.020 (−0.004, 0.044)	0.101	0.368
Antisaccade costs [ms]	−0.474 (−1.760, 0.812)	0.470	0.620	−0.531 (−1.806, 0.744)	0.414	0.665	−0.526 (−1.804, 0.751)	0.419	0.564
Antisaccade error rate [%]	0.152 (−0.491, 0.794)	0.644	0.751	0.243 (−0.398, 0.885)	0.457	0.665	0.298 (−0.344, 0.941)	0.363	0.564

The table displays the change in cognitive performance per one standard deviation increase in Alzheimer’s disease PRS for the three different PRS scores separately. The regression coefficients for each PRS were obtained from the following multivariable linear regression model: Cognitive outcome ~ b0 + PRS* b1 + age + age^2^ + sex + population stratification + residual error. The FDR-correction is based on 28 comparisons (27 in the table plus antisaccade correction rate) and was conducted for each PRS score separately. None of the association between PRS and cognitive performance remained significant after excluding *APOE* from the PRS. In bold are those associations with an unadjusted *p*-value below 0.05.

*AD* Alzheimer’s disease, *b* unstandardized regression coefficient, *FDR* false discovery rate, *AVLT* Auditory Verbal Learning and Memory Test, *MWT-B* Mehrfachwahl-Wortschatz-Intelligenztest, 95%-CI = 95%-confidence interval.

Higher genetic risk for AD was significantly associated with lower performance in the Corsi forward task across all three AD PRS scores but these associations did not remain significant after correction for multiple testing. Additionally, before correcting for multiple testing, a higher PRS_Jansen_ score was associated with lower saccade frequency in the smooth pursuit task, higher prosaccade latency, and a lower probability of correcting all antisaccade direction errors (odds ratio and 95% confidence interval (OR and 95%-CI): 0.884 (0.810–0.964); *p* = 0.005; FDR-adjusted *p* = 0.140), but not with the proportion of uncorrected errors in those participants who did not correct all of their direction errors (OR and 95%-CI: 0.997 (0.943–1.054); *p* = 0.908). The uncorrected *p*-value also indicated that PRS_Schwartzentruber_ was associated with a lower probability of correcting all antisaccade direction errors (OR and 95%-CI: 0.916 (0.840–0.999); *p* = 0.047; FDR-adjusted *p* = 0.235), but not with the percentage of uncorrected direction errors in those who did not correct all of their antisaccade direction errors (OR and 95%-CI: 0.990 (0.935–1.048); *p* = 0.727). As for the other two PRS, only the uncorrected *p*-values indicated that a higher PRS_Wightman_ score was associated with better performance in the digit span backward and lower TMT-A performance. PRS_Wightman_ was neither associated with the probability of correcting all versus not all antisaccade direction errors (OR and 95%-CI: 0.920 (0.844–1.003); *p* = 0.058), nor with the percentage of uncorrected errors in those who did not correct all of their antisaccade errors (OR and 95%-CI: 0.988 (0.933–1.047); *p* = 0.685).

Exclusion of AD cases (*N* = 5, Table 1) from the sample, or adding educational level as an additional covariate, did not materially change the results (data not shown).

### Interaction effects

**Fig. 1:**
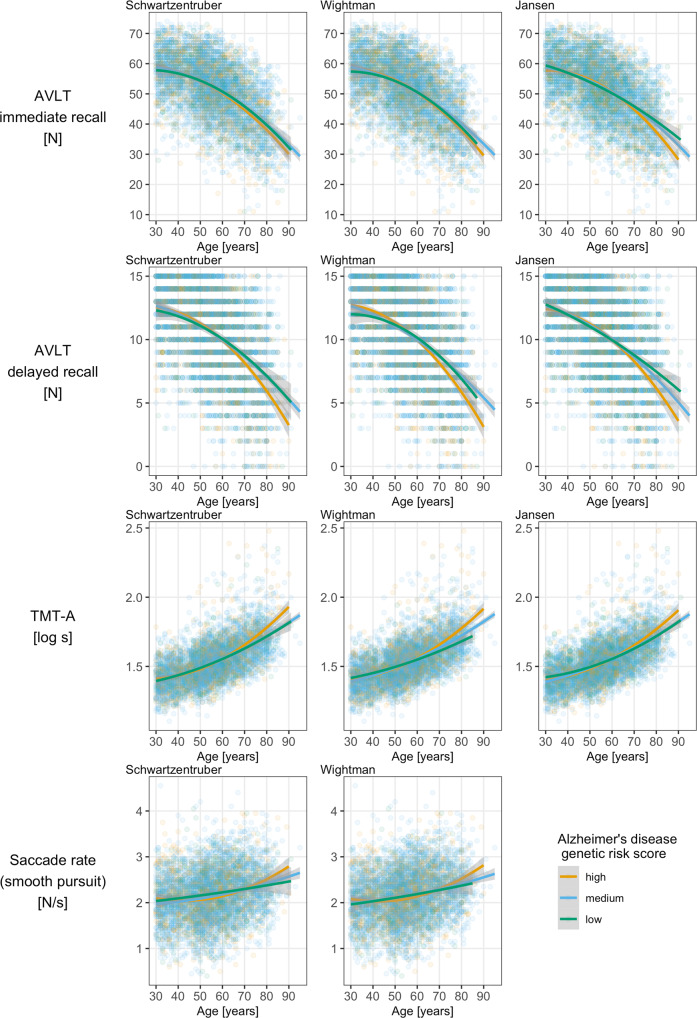
Scatterplots for interaction effects between age and Alzheimer’s disease polygenic risk scores (PRS). The scatterplots show how the associations between age and different cognitive outcomes vary with genetic risk for Alzheimer’s disease. Each column represents a different polygenic risk score and each row represents a cognitive outcome. The colours represent three different genetic risk groups for Alzheimer’s disease (orange = high risk/ z-standardized PRS score above 1; blue = medium risk/ z-standardised PRS score between −1 and 1; green = low risk/ z-standardised PRS score below −1). For each genetic risk group there exists one superimposed function for the development of the cognitive outcome across the adult life span. The functions were obtained from a multivariable regression model with the following formula: cognitive outcome ~ b_0_ + age*b_1_ + age^2^*b_2_ + residual error. The grey area around the risk group-specific regression lines indicates the 95% confidence interval in each case. AVLT Auditory Verbal Learning and Memory Test; TMT-A Trail-making test A, N = number.

We found significant interactions between the three different PRS and age and age^2^ for TMT-A performance that remained significant after correcting for multiple testing (Table 3). For AVLT (immediate and delayed recall), the interactions between PRS and age and age^2^ were also significant for all three different PRS, but only the interactions between PRS_Jansen_ and age and age^2^ for AVLT immediate recall remained significant after correcting for multiple testing. In addition, we found significant interaction effects between PRS_Wightman_, and PRS_Schwartzentruber_ and age and age^2^ for saccade frequency during smooth pursuit, but they did not survive correction for multiple testing. Visualisation of the interaction effects showed that individuals with the highest genetic risk for AD showed the strongest age-related decline in AVLT (immediate and delayed recall) and TMT A performances (Fig. 1). For saccade frequency during smooth pursuit, the scatterplot did not reveal a clear pattern (Fig. 1). Post-hoc comparisons using the Tukey test revealed that delayed recall performance in individuals at highest genetic risk, based on PRS_Wightman_ and PRS_Schwartzentruber_ was worse compared to those in the medium (PRS_Wightman_ model: *p* = 0.001, PRS_Schwartzentruber_ model: *p* = 0.025) and low (PRS_Wightman_ model: *p* = 0.017, PRS_Schwartzentruber_ model: *p* = 0.031) genetic risk groups. Additionally, high genetic risk individuals performed worse than medium (PRS_Wightman_ model: *p* = 0.018, PRS_Schwartzentruber_ model: *p* = 0.016) and low (PRS_Wightman_ model: *p* < 0.001, PRS_Jansen_ model: *p* = 0.003) genetic risk individuals in TMT-A performance. Further, in the PRS_Wightman_ model, medium genetic risk individuals differed from low genetic risk individuals in TMT-A performance (*p* = 0.016). All other post-hoc comparisons were non-significant.

**Table 3:** Testing for interactions between Alzheimer’s Disease PRS and age and age^2^ on cognitive performance.

Cognitive outcome	*F*-value in model with PRS Jansen	*p*-value	FDR-adjusted *p*-value	*F*-value in model with PRS Wightman	*p*-value	FDR-adjusted *p*-value	*F*-value in model with PRS Schwartzentruber	*p*-value	FDR-adjusted *p*-value
Outcomes - classical cognitive tasks									
Digit span forward [number of digits]	2.174	0.114	0.579	2.344	0.071	0.320	2.116	0.096	0.432
Digit span backward [number of digits]	1.898	0.150	0.579	1.304	0.271	0.617	1.280	0.279	0.731
Corsi forward [number of blocks]	1.164	0.312	0.782	1.295	0.274	0.617	1.028	0.379	0.731
Corsi backward [number of blocks]	1.222	0.295	0.782	1.071	0.360	0.730	1.101	0.347	0.731
AVLT – immediate recall [sum of recalled words]	**5.780**	**0.003**	**0.042**	**4.256**	**0.005**	**0.070**	**4.125**	**0.006**	**0.084**
AVLT – delayed recall [number of words]	**5.091**	**0.006**	**0.056**	**3.526**	**0.014**	**0.097**	**3.507**	**0.015**	**0.099**
Word fluency task [number of animals]	0.501	0.606	0.877	0.684	0.562	0.844	0.541	0.654	0.921
Trail-making test A [log s]	**10.165**	**<0.001**	**0.001**	**7.046**	**<0.001**	**0.003**	**6.976**	**<0.001**	**0.003**
Trail-making test B [log s]	2.163	0.115	0.579	1.631	0.180	0.540	1.634	0.179	0.589
MWT-B [sum of correctly recognised words]	1.423	0.241	0.782	1.029	0.379	0.730	1.060	0.365	0.731
Outcomes - eye movement tasks									
Log of spatial error during fixation [log °]	0.266	0.766	0.877	0.288	0.834	0.925	0.274	0.844	0.925
Log of saccade rate during fixation [log N/s]	0.700	0.496	0.877	0.584	0.625	0.844	0.779	0.505	0.853
Log of blink rate during fixation [log N/s]	0.388	0.679	0.877	0.632	0.594	0.844	1.076	0.358	0.731
Smooth pursuit velocity gain [%]	0.026	0.974	0.974	0.129	0.943	0.943	0.158	0.925	0.925
Saccade frequency during smooth pursuit [N/s]	2.000	0.135	0.579	**3.733**	**0.011**	**0.097**	**3.551**	**0.014**	**0.099**
Prosaccade latency [ms]	1.144	0.319	0.782	2.393	0.067	0.320	1.876	0.131	0.507
Prosaccade amplitude gain [%]	1.018	0.361	0.813	0.679	0.565	0.844	0.710	0.546	0.867
Log of prosaccade spatial error [log %]	0.445	0.641	0.877	0.619	0.603	0.844	0.393	0.758	0.925
Prosaccade peak velocity [°/s]	0.159	0.853	0.886	1.348	0.257	0.617	0.870	0.456	0.820
Amplitude-adjusted peak prosaccade velocity	0.820	0.440	0.877	1.965	0.117	0.395	1.562	0.196	0.589
Antisaccade latency [ms]	0.331	0.718	0.877	0.618	0.603	0.844	0.500	0.682	0.921
Antisaccade amplitude gain [%]	0.403	0.669	0.877	0.290	0.833	0.925	0.386	0.763	0.925
Log of antisaccade spatial error [log %]	0.227	0.797	0.877	2.184	0.088	0.338	2.229	0.083	0.432
Antisaccade peak velocity [°/s]	0.208	0.812	0.877	0.257	0.856	0.925	0.174	0.914	0.925
Amplitude-adjusted peak antisaccade velocity	0.270	0.764	0.877	0.168	0.918	0.943	0.179	0.911	0.925
Antisaccade costs [ms]	0.397	0.672	0.877	0.305	0.821	0.925	0.302	0.824	0.925
Antisaccade error rate [%]	0.757	0.469	0.877	0.515	0.672	0.864	0.529	0.662	0.921

The table displays the results of the likelihood ratio test, which compares the model fit of model 1 (cognitive variable ~ b_0_/β_0_ + PRS* b_1_/ β_1_ + age + age^2^ + sex + residual error) with the model fit of model 2 (cognitive variable ~ b_0_/β_0_ + PRS*b_1_/ β_1_ + age*PRS*b_3_/β_3_ + age^2^*PRS*b_4_/β_4_ + age + age^2^ + sex + residual error). The null hypothesis says that the data are equally likely under both models and can be rejected if the p-value is <0.05; otherwise, it cannot be rejected. Interpretation of the *F*-value: the data are “*F*-value” times more likely if the interaction terms “age*PRS” and “age^2^*PRS” are included in the model (model 2) than if they are not included in the model (model 1). We conducted the likelihood ratio tests for each PRS score separately and present the resulting *F*-values and corresponding *p*-values and FDR-adjusted *p*-values next to each other. The FDR-correction is based on 27 comparisons. In bold are those associations with an unadjusted *p*-value below 0.05.

*PRS* polygenic risk score, *SNP* single-nucleotide polymorphism, *FDR* false discovery rate, *AVLT* Auditory Verbal Learning and Memory Test.

### Statistical power analysis

Our post-hoc analysis showed that we could detect effect sizes (Cohen’s f^2^) of 0.0020, 0.0015 and 0.0010 with a statistical power of 90%, 80% and 62%, respectively. To illustrate the magnitude of the effect sizes that we were able to detect, we calculated the effect sizes for the associations between the three different AD PRS scores and Corsi forward performance. The effect sizes were f^2^ = 0.0011 for PRS_Jansen_, f^2^ = 0.0010 for PRS_Wightman_ and f^2^ = 0.0016 for PRS_Schwartzentruber_.

## Discussion

We found that genetic risk for AD was not significantly associated with any cognitive or oculomotor measure from our test battery after correcting for multiple testing. However, prior to correcting for multiple testing, all three AD PRS were significantly associated with lower performance in the Corsi forward working memory task, and two AD PRS with a lower probability of correcting versus not correcting all antisaccade errors. Further, the association between age and TMT-A performance varied with genetic risk for AD and was strongest in those individuals at highest genetic risk for AD.

The unadjusted *p*-values indicated an association between all three AD PRS and visuo-spatial working memory performance as measured by the Corsi forward task, which has not been found in previous studies [33, 37]. We found that the associations between AD PRS and Corsi forward performance did not vary with age, suggesting that the discrepancy between our and previous studies is unlikely to be due to differences in the age distribution. One reason for this finding may be that we created the PRS based on more recent genome-wide association studies and, therefore, included at least seven more SNPs in our PRS than previous studies. However, a previous study comparing visuo-spatial working memory performance between homozygous *APOE* ε4 carriers and non-ε4 carriers reported lower Corsi performance (combined score for forward and backward performance) in homozygous *APOE* ε4 carriers [56]. Still, the associations between AD PRS and Corsi forward performance did not remain significant after correcting for multiple testing. FDR-correction probably was too conservative as discussed further below this. Nevertheless, our results should be considered as suggestive until validated in independent studies. Concerning the other tests for visuo-spatial working memory, we found that a higher PRS_Wightman_ score was associated with a *better* digit span backward performance. However, this association was not found using PRS_Jansen_ and PRS_Schwartzentruber_, thus likely representing a false positive observation.

Regarding associations between genetic risk for AD and episodic verbal memory performance, previous studies have reported significant associations [31–33], which we could not confirm across the entire range of 30+ year-olds. However, our results suggest that the association between age and AVLT immediate recall varied with genetic risk for AD (Table 3), and was strongest in those individuals at highest genetic risk for AD (Fig. 1). For PRS_Jansen_, this interaction effect remained significant after correction for multiple testing. Additionally, the association between age and AVLT delayed recall was also modified by genetic risk for AD, with AVLT delayed recall declining strongest in those individuals at highest genetic risk for AD, as indicated by Fig. 1 and the results of Tukey post-hoc tests for PRS_Wightman_ and PRS_Schwartzentruber_. However, these interactions did not remain significant after correction for multiple testing. Thus, our findings overall suggest that differences in episodic verbal memory performance among the three genetic risk groups may become more pronounced at older ages, which is compatible with the finding that age is a major risk factor for AD [53].

Associations between all three PRS scores and TMT-A performance varied robustly with age, as the interaction terms remained significant even after correcting for multiple testing. We observed that differences in TMT-A performance among the three PRS groups were strongest at older ages (Fig. 1). Across the entire age range, only PRS_Wightman_ was associated with lower TMT-A performance, but this association did not remain significant after correction for multiple testing. A previous population-based study reported no association between AD PRS and processing speed across the sample but only in the 70- to 99-year-olds [31]. Thus, our results support the previous finding that associations between AD PRS and TMT-A performance are more likely to emerge in old age. Additionally, our robust finding that genetic risk for AD gradually affects the magnitude of age-related decline in processing speed, but not in other cognitive domains across the adult lifespan, supports Salthouse’s processing speed theory of age-related cognitive decline [57]. According to this theory, slowing of processing speed is the global mechanism underlying age-related cognitive decline [57]. This suggests that AD partly results from individuals at high genetic risk for AD experiencing a stronger age-related decline in processing speed compared to individuals at low genetic risk for AD, resulting in lower cognitive performance across all domains in the long term. Our finding is also in line with previous reports of AD PRS being related to longitudinal decline in processing speed [34, 35].

Consistent with some previous studies [32, 37], we found no associations between AD PRS and executive function, as measured by performance in TMT-B and the word fluency task. Associations between AD PRS and oculomotor measures had not been assessed before. Using AD PRS_Jansen_, we found that higher genetic risk was associated with higher prosaccade latency, lower saccade frequency during pursuit, and lower antisaccade error correction probability. However, as neither of those associations could be found with the other two AD PRS scores and as none of the findings survived correction of multiple testing, they should be interpreted with caution as they may represent false positive findings.

Our finding of a lower probability of correcting antisaccade errors in individuals with higher genetic risk for AD may be more robust as it was found using both PRS_Jansen_ and PRS_Schwartzentruber_. Still, neither association remained significant after correction for multiple testing. The association between genetic risk for AD and a lower probability of correcting antisaccade errors agrees with previous reports of lower antisaccade error correction probabilities in individuals with AD and MCI [13, 15, 16]. Moreover, scores in dementia screening tests have also been found to correlate with the probability of correcting antisaccade errors [24]. Still, the association between genetic risk for AD and antisaccade corrections probability requires further investigation as it was not found using PRS_Wightman_ and did not survive correction for multiple testing.

A potential limitation of our study is lack of longitudinal data, precluding assessment of the associations between AD PRS and change in cognitive outcomes. However, our sample included a wide age range, which allowed us to investigate associations between AD PRS and cognitive outcomes across the adult lifespan, and how the associations between age and cognitive tests vary between different AD genetic risk groups. Further, we employed an extensive cognitive test battery including eye movement outcomes that were not part of the cognitive test batteries in previous large population studies.

Another potential limitation of our study relates to statistical power. The associations between AD PRS and cognitive measures did not remain significant after FDR-correction. However, our approach for correcting for multiple comparisons may have been too conservative as it is only appropriate in case of disjunction testing [58]. On the one hand, we wanted to infer from the individual cognitive measures which cognitive domain is most sensitive to capturing genetic risk for AD. In this scenario, one could argue that our testing approach represents disjunction testing, as a significant association between genetic risk and a cognitive measure would be taken as an indication that the represented cognitive domain in general is especially sensitive to capturing genetic risk for AD [58]. On the other hand, we aimed to identify the most sensitive *individual* cognitive measure of genetic risk for AD without making a general statement about the associated cognitive domain. This approach would clearly fall into the category of individual testing, for which correction for multiple testing is not appropriate [58]. Thus, as long as we do not overgeneralize from single tests to cognitive domains, correction for multiple testing may be too conservative. Importantly, we also conducted the analyses using three different AD PRS scores to identify the most consistent associations. Moreover, it should be noted that current AD PRS only explain about 7% of AD heritability at optimal *p*-value threshold for SNP inclusion [44], despite heritability estimates for AD ranging from 58% to 79% [59]. This may be due to PRS being based on summary statistics of GWAS that rely on conventional genotyping arrays that capture common variants but not rare or structural variants [29]. Finally, AD is a heterogeneous disease and influenced by a complex interplay between both genetic and environmental factors [60]. Thus, very strong associations between AD PRS and cognitive outcome are unlikely to occur, exemplified by the small effect sizes (Cohen’s f^2^ ≤ 0.0016) for the associations between the three different AD PRS scores and Corsi forward performance. Therefore, Corsi forward performance and the likelihood of correcting antisaccade errors may be promising candidate measures whose ability to capture the genetic predisposition to AD should be investigated further in future studies. Lastly, the suitability of TMT-A performance in detecting genetic liability for AD in old age also requires further investigation.

## Conclusion

PRS for AD were not robustly associated with any of our cognitive and oculomotor measures. Of all our cognitive measures, Corsi forward performance and the probability of correcting antisaccade errors may be the most suitable candidates for capturing genetic liability for AD across the adult lifespan, but these associations require confirmation in independent samples. In addition, TMT-A, which measures processing speed performance, may be a sensitive marker of genetic susceptibility to AD in old age. Lastly, our finding of a stronger association between age and processing speed performance in individuals at high genetic risk for AD supports the processing speed theory of age-related cognitive decline by Salthouse, suggesting a decline in processing speed as the global mechanism underlying general cognitive decline.

## Data Availability

The datasets for this manuscript are not publicly available because of data protection regulations. Access to data can, however, be provided to scientists in accordance with the Rhineland Study’s Data Use and Access Policy. Requests to access the datasets should be directed to Dr Monique Breteler, RS-DUAC@dzne.de.
